# Amorphous Silicon p-i-n Structure Acting as Light and Temperature Sensor

**DOI:** 10.3390/s150612260

**Published:** 2015-05-26

**Authors:** Giampiero de Cesare, Augusto Nascetti, Domenico Caputo

**Affiliations:** 1 Department of Information Engineering, Electronics and Telecommunications, “La Sapienza” University of Rome, via Eudossiana 18, 00184 Rome, Italy; E-Mail: caputo@diet.uniroma1.it; 2 Department of Astronautics, Electrical and Energetic Engineering, “La Sapienza” University of Rome, via Eudossiana 18, 00184 Rome, Italy; E-Mail: augusto.nascetti@uniroma1.it

**Keywords:** temperature sensors, photosensors, amorphous silicon devices, Lab-on-Chip

## Abstract

In this work, we propose a multi-parametric sensor able to measure both temperature and radiation intensity, suitable to increase the level of integration and miniaturization in Lab-on-Chip applications. The device is based on amorphous silicon p-doped/intrinsic/n-doped thin film junction. The device is first characterized as radiation and temperature sensor independently. We found a maximum value of responsivity equal to 350 mA/W at 510 nm and temperature sensitivity equal to 3.2 mV/K. We then investigated the effects of the temperature variation on light intensity measurement and of the light intensity variation on the accuracy of the temperature measurement. We found that the temperature variation induces an error lower than 0.55 pW/K in the light intensity measurement at 550 nm when the diode is biased in short circuit condition, while an error below 1 K/µW results in the temperature measurement when a forward bias current higher than 25 µA/cm^2^ is applied.

## 1. Introduction

Lithography-based microtechnology, initially used to realize integrated semiconductor structures for microelectronic chips, was soon applied to fabricate microsystem devices suitable for several applications. In particular, the introduction of the concept of the micro Total Analysis Systems (µTAS) [1] and the development of the Micro Electro Mechanical Systems (MEMS) led to the development of Lab-on-Chip (LOC) systems as a powerful tool for biomolecular analysis in point-of-care applications [2,3,4,5]. LOC device is an example of a system where the high miniaturization level allows accomplishing several laboratory functions, usually done at lab-scale, with a fast response time, low sample consumption and on-site operation [6,7]. Although the application of LOCs is still novel and modest, a growing interest of companies and research groups is observed in different fields, such as chemical analysis, environmental monitoring, and medical diagnostics. However, only few LOC are currently commercially available and in many cases laboratory equipment is still required for their operation. Thus, current research efforts are directed towards the integration, on the same substrate (that can be either glass, plastic or crystalline silicon), of different physical and optical sensors, together with microfluidic devices, in order to produce a single analysis tool, where all the steps of the analysis, such as sample preparation, sample handling and analytical detection, can be carried out [8,9,10].

Sample preparation is usually implemented by heating the substrate for thermal treatments of the biomolecules and/or to improve the surface functionalization [11,12]. Monitoring and control of temperature is often performed with thin film sensors due to their high degree of integration [13].

Detection is usually performed using off-chip detection systems able to measure the fluorescence emitted by fluorescent dyes attached to the target molecules [14]. Recently, different groups have integrated electrical and/or optical sensors with the microfluidics, with the aim of achieving on-chip detection for improving the system sensitivity and compactness. In particular, optical detection of biomolecules based on organic [15,16,17] and inorganic [18] thin film photosensors has been developed.

One of the most promising materials to this aim is hydrogenated amorphous silicon (a-Si:H) and its alloy. The low deposition temperature (below 250 °C) and its physical characteristics prompt the use of this material in different devices such as solar cells [19], electronic switching [20], strain sensors [21,22] and photosensors [23]. The use of thin film a-Si:H photosensors for the detection of biomolecules has already been developed by different research groups [24,25,26,27,28,29], and in particular by the authors in both labeled and label free techniques [30,31,32]. Furthermore, the same a-Si:H structure has recently been used as temperature sensor in a lab-on-glass system for the molecular amplification by Polymerase Chain Reaction (PCR) technique, in order to achieve a point-monitoring of the temperature distribution within the device area [33,34,35].

In this work, we propose the use of a single a-Si:H diode as multi-parametric sensor to measure both temperature and radiation intensity, in order to increase the level of integration and the miniaturization of LOC systems. In particular, we present a detailed investigation of the requirements that the structure has to satisfy, taking into account that the temperature variation affects the photo-response and that, on the other hand, light intensity variation can induce a reduction of accuracy in the temperature measurement. 

The paper is organized as follows: details of the device fabrication process are reported in the Section 2 “Materials and Methods”, in Section 3 the structure and the fabrication process of the a-Si:H device are described, together with the characterization of the structure as radiation and temperature sensor; in Section 4, experimental results are described and discussed in details with focus on the reciprocal influence of the two measured parameters; and in Section 5, conclusions are drawn..

## 2. Material and Methods

### Fabrication Process of the Device

We have fabricated several 2 × 2 mm^2^ diodes arranged in array structure by the use of standard microelectronic technologies and four photolithographic steps for the geometry patterning.

Below are described details of the technological steps used for the fabrication of the diode array.

1. Cleaning of the glass substrate:
  - ultrasonic cleaning in Detergent 8 (by Alconox) diluted at 5% in deionized water (DI) for 30 min;
  - ultrasonic rinse in DI at 50 °C for 15 min;
  - ultrasonic cleaning in Liquinox (by Alconox) diluted at 2% in DI for 30 min;
  - ultrasonic rinse in DI at 50 °C for 15 min;
  - drying in nitrogen flow; and
  - immediately positioning of the glass substrate inside the RF magnetron sputtering system.
2. Sputter deposition (in Material Research Corporation system) of 200 nm thick layers from a 6-in-diameter Indium Tin Oxide (ITO) target (with 90% In_2_O_3_–10% SnO_2_ in weight composition), at 200 W of RF power, 2.7 mTorr of pressure process, 25 sccm of argon flow and 120 °C substrate temperature (Figure 1a).
3. First lithographic step:
  - spin coating of photoresist (AZ1518 from Shipley);
  - soft bake at 100 °C for 1 min;
  - UV exposure through a lithographic mask at 320 mJ/cm^2^ for 45 s; and
  - development of the photoresist in AZ351B diluted at 25% in DI for 45 s.
4. Patterning of ITO layer (with mask #1 in Figure 1b) by Sputter Etching at 180 W, 50 sccm Argon flow and 90 mTorr.

**Figure 1:**
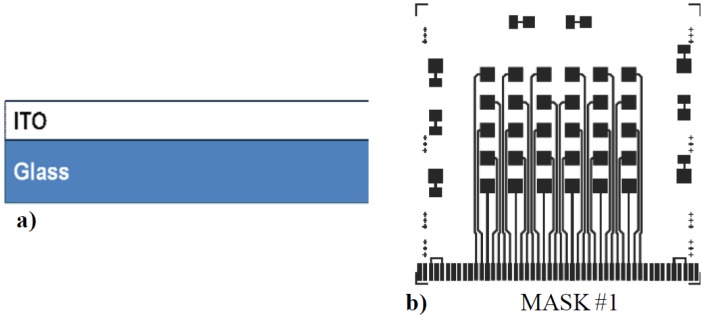
(**a**) Structure of the sensor after step 4 of the fabrication process. (**b**) Photolithographic mask used for the patterning of ITO bottom electrode of the device.
5. Covering the ITO markers with kapton tape to allow the subsequent alignments.
6. Sequential deposition, by Plasma Enhanced Chemical Vapor Deposition (PECVD) in a three UHV chamber system, of the hydrogenated p-type a-SiC, intrinsic and n-type a-Si layers, that constitute the stacked structure of the diode. The following Table 1 reports the deposition parameters of the PECVD process.

**Table 1:** PECVD parameters used in the deposition of the a-Si:H and a-SiC:H layers. The gases are: SiH_4_ pure silane, PH_3_ silane diluted (5%), B_2_H_6_ helium diluted (5%), CH_4_ pure methane; P_D_ is the process pressure; P_RF_ is the power density of the plasma discharge; T_D_ is the substrate temperature; t_D_ is the deposition time. The thicknesses reported above have been estimated from the growth rate of the different materials.

Layer type/material	SiH_4_ (sccm)	PH_3_ (sccm)	B_2_H_6_ (sccm)	CH_4_ (sccm)	P_D_ (Torr)	P_RF_ (mW/cm^2^)	T_D_ (°C)	t_D_ (sec)	Thickness (nm)
p/a-SiC:H	40		5	60	0.7	25	150	60	10
i/a-Si:H	40				0.68	25	180	2100	400
n/a-Si:H	40	10			0.3	25	200	180	50
7. Deposition of a three metal layer stack of Cr/Al/Cr with thickness of 30/200/30 nm, respectively, in an Ultra Vacuum system (thermal evaporator by Balzers) with measured growth rate of 0.3 nm for Cromium and 1 nm for Aluminum.
8. Peeling of the kapton tape, for the subsequent alignments.
9. Lithographic steps (see point 3) with Mask #2 in Figure 2b.
10. Patterning of the Cr layer by wet etching in a solution of 30 g Ce(NH_4_)_2_(NO_3_)_6_, 9 mL CH_3_COOH and 200 mL DI for 1 min.
11. Patterning of the Aluminum layer by wet etching in a solution of 80 mL H_3_PO_4_ (85%), 5 mL HNO_3_ and 10 mL DI.
12. Patterning of the Cr layer by wet etching (see point 10).
13. Patterning of the a-Si:H n-i-p structure by dry etching in a Reactive Ion Etching system (by IONVAC) (Figure 2a) with the following parameters:
  - Oxigen (O_2_) flow O_2_: 10 sccm;
  - Carbon tetrafluoride (CF_4_) flow: 100 sccm; and
  - Radio Frequency Power Density: 300 mW/cm^2^.

**Figure 2:**
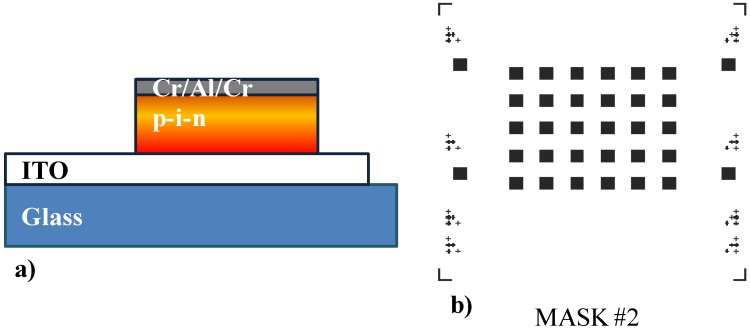
(**a**) Structure of the sensor after the step 13 of the fabrication process. (**b**) Photolithographic mask used for the patterning of the mesa structure of the device.
14. Deposition by spin coating of a 5 µm-thick SU8 (from Micro-Chem, MA, USA) and its pattering through Mask #3 in Figure 3b for opening via holes over the diodes (Figure 3a). The deposition of the SU-8 film is implemented through the following steps:
  - spin coating at 500 rpm for 5 s followed by another run at 3000 rpm for 30 s;
  - bake at 65 °C for 1 min followed by another bake at 95 °C for 2 min;
  - UV-light exposition at 250 mJ/cm^2^;
  - bake at 65 °C for 1 min followed by another bake at 95 °C for 1 min;
  - developing in SU-8 remover;
  - rinse in isopropyl alcool; and
  - hard bake at 150 °C for 30 min.

**Figure 3:**
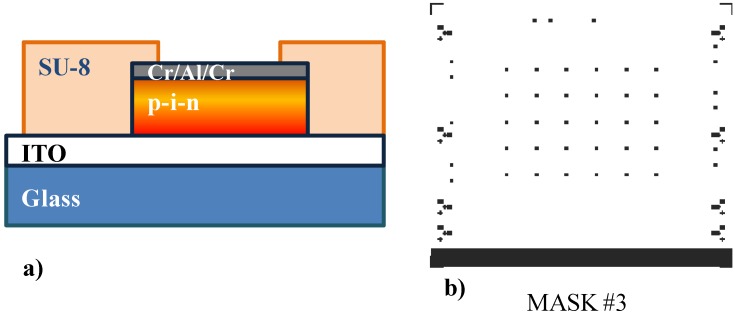
(**a**) Structure of the sensor after step 14 of the fabrication process. (**b**) Photolithographic mask used for the patterning of the via on the SU8 insulation layer.
15. Deposition of a three metal layer stack of Cr/Al/Cr with thickness of 30/200/30 nm, respectively, in an Ultra Vacuum system (see point 7).
16. Lithographic steps (see point 3) with Mask #4 in Figure 4b.
17. Patterning of the Cr/Al/Cr layers (see points 10 an 11).

**Figure 4:**
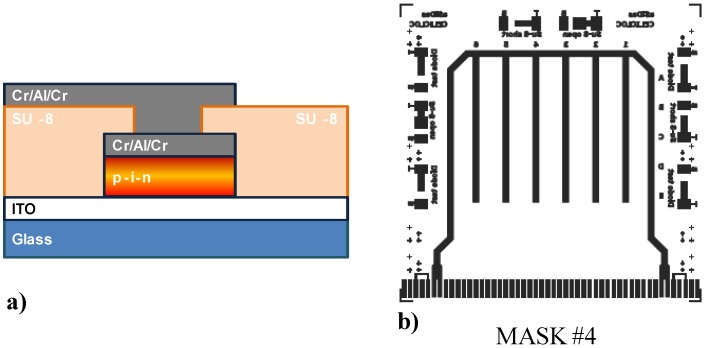
(**a**) Structure of the sensor after step 17 of the fabrication process; (**b**) Photolithographic mask used for the patterning of the metallic lines for the electrical connection of the top electrode of the device to the edge of the glass substrate.
18. Deposition by spin coating of a 5 µm-thick SU-8 as passivation layer and hard baking at 150 °C for 30 min.

## 3. Structure and Characterization of the Device

### 3.1. Sensor Structure

The basic structure of the device is a p-type doped amorphous silicon carbide (a-SiC:H)/intrinsica-Si:H/n-type doped a-Si:H heterojunction. The diode is deposited by Plasma Enhanced Chemical Vapor Deposition (PECVD) on a glass substrate covered with a Indium Tin Oxide (ITO) transparent conductive layer [36]. This layer acts as bottom electrode of the device, and for the light sensor application, as window layer for the light impinging on the photodetector through the glass. A Cr/Al/Cr stacked structure vacuum evaporated on the a-Si:H diode behaves as the top contact of the structure. An SU-8 insulation layer is deposited over the diode in order to avoid the short circuit between top and bottom electrodes and to reduce the current component induced by or the exposed diode perimeter [37].

We have fabricated several 2 × 2 mm^2^ diodes, arranged in array structure, by the use of standard microelectronic technologies: Physical and Chemical Vapor Deposition of thin film, Dry and Wet etching of different materials, and four photolithographic steps for the geometry patterning. All the details of the device fabrication process are reported in Section 5 “Material and Methods”.

The device current-voltage characteristics were evaluated using a Keithley 236 Source Measure Unit (SMU). We achieved a current of 10^−11^ A/cm^2^ at small (10 mV) reverse voltage, with an excellent reproducibility among the samples. This value determines a noise current contribution in the order of 2 fA/√Hz, which is below the minimum detectable signal in our experimental set-up.

### 3.2. Characterization as Radiation Sensor

Due to the a-Si:H optical properties, the p-i-n junction is widely used as thin film devices for light detections. The active layer of the sensor is the intrinsic one, where the holes/electrons, photo-generated by the absorbed light, are swept toward the doped-regions by the electric field existing in the intrinsic region. The doped layers provide the built-in potential of the junction, but do not contribute to the photocurrent, because the high density of charged dangling bond defects, induced by doping in amorphous silicon, strongly traps the minority photo-generated carriers [37].

The spectral responsivity of the p-i-n photodiode can be designed through the optical absorption of the three layers, by tuning their thicknesses and energy gaps. In our devices, the energy gap of the p, i and n-type materials, measured on single film deposited on Corning glass substrates, are equal to 1.92, 1.79 and 1.71 eV, respectively, while the thicknesses are equal to 10, 400 and 50 nm, respectively.

The structure has been designed with the aim to maximize the responsivity at wavelengths between 400 and 600 nm, which ensures the suitability of our photosensors for biochemical applications, where chemiluminescent or fluorescent signals have to be detected [38,39,40].

**Figure 5:**
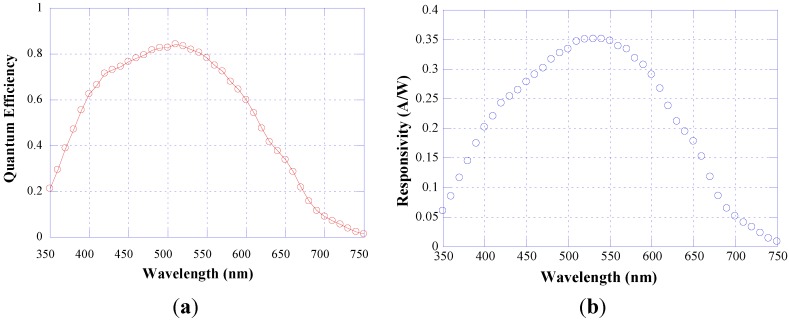
Quantum efficiency (**a**) and responsivity (**b**) curves of p-i-n a-Si:H photodiode measured in short circuit condition.

Figure 5a reports the quantum efficiency (QY) curve of one photodiode, measured in short circuit condition. As reported in [41], this operation mode minimizes the peripheral leakage current and therefore this is the bias condition we have chosen when the diode acts as radiation sensor. Characterization has been performed on a double-arm set-up, including a tungsten light source, a monochromator Spex 340E, a beam splitter and a calibrated UV-enhanced crystalline silicon diode.

From this characterization and from the following equation:

(1) $$R=\frac{QY\cdot \lambda \cdot q}{hc}$$

where *λ* is the wavelength, *q* the electron charge, *h* the Planck constant and *c* the light speed, we derive that the photosensor responsivity (*R*) reported in Figure 5b. *R* is around 280 mA/W at 450 and 600 nm and shows a maximum equal to 350 mA/W at 510 nm.

### 3.3. Characterization as Temperature Sensor 

**Figure 6:**
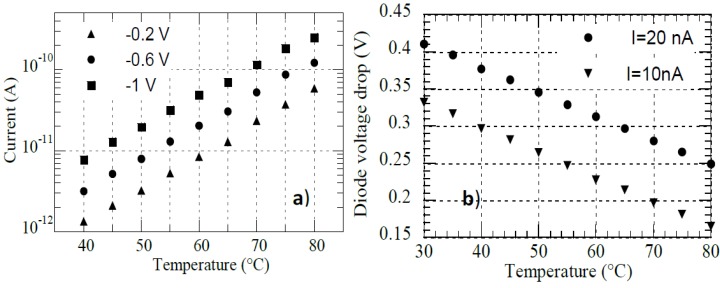
(**a**) Current flowing in the device diode as a function of temperature at reverse voltage bias equal to −0.2 V (triangle symbols), −0.6 V (circular symbols) and −1 V (square symbols); (**b**) Voltage drop across the diode as a function of temperature when a forward current of 10 nA (triangle symbols) or 20 nA (circular symbols) is flowing in the device.

The same a-SiC:H/a-Si:H p-i-n diodes have been characterized as temperature sensors measuring their current–voltage characteristics as a function of temperature in the range 30 °C–90 °C, both under forward and reverse bias voltage conditions. Measurements have been performed in a probe station with a temperature-controlled chuck, by using a thermocouple as temperature reference. As expected [42], at constant voltage, in reverse bias condition, we observe an exponential dependence of the reverse saturation current with temperature, while at constant current, in forward bias condition, a linear decrease of the voltage across the diode with temperature results. These results are summarized in Figure 6. In particular, Figure 6a reports the diode current as a function of temperature when the diode is biased at 0.2, 0.6 or 1 V reverse voltage, while Figure 6b shows the voltage drop across the diode as a function of temperature when a forward current of 10 or 20 nA is flowing in the device.

In forward bias condition, with a bias current higher than 10 nA, the achieved temperature sensitivity (*S_T_*) is equal to 3.2 mV/K, greater than the one of a crystalline silicon diode [43], showing that the a-Si:H diode can be considered a very promising candidate for detecting very small temperature variations in LOC applications.

## 4. Results and Discussion

The use of the same device as both light and temperature sensor has to consider the mutual influence of these two physical parameters. Indeed, temperature variation affects the current flowing through an irradiated diode, and, on the other hand, light intensity variation can induce a reduction of accuracy in the temperature measurement. In the following, we analyze in some detail the effect of one parameter (light intensity or temperature) on the measurement of the other parameter (temperature or light intensity, respectively).

### 4.1. Temperature Effect on Light Measurement

**Figure 7:**
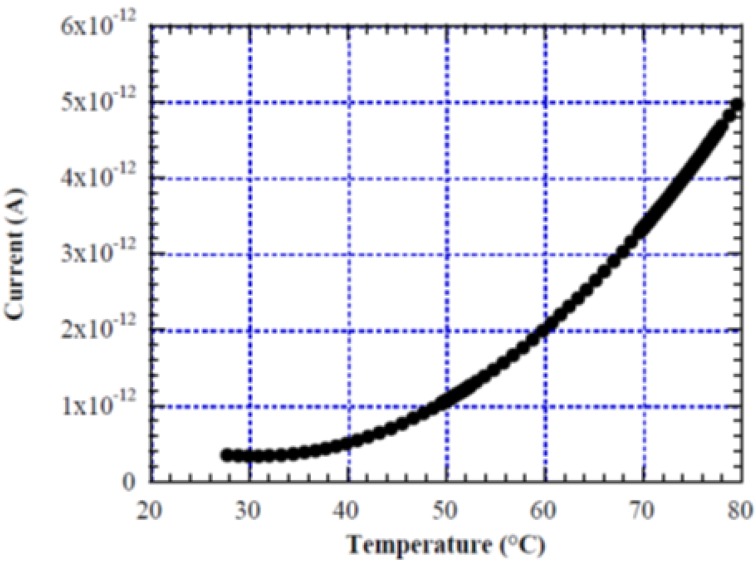
Diode dark current as function of temperature, with the device biased to the virtual ground of the amplifier.

To investigate the effect of temperature on the sensor photo-response, we measured the current flowing in the a-Si:H diode as a function of temperature. In particular, Figure 7 reports the diode current, measured through a charge sensitive circuit with the device in dark condition and biased to the virtual ground of the amplifier, in the temperature range 25–80 °C.

We see an increase of the dark current (*I_D_*) from 0.25 pA at room temperature to 5 pA at 80 °C. The slope of this curve increases exponentially with temperature, with a maximum of 195 fA/K.

When the device is used as light sensor the output signal is a current proportional to the light intensity. In this case, a current variation (*ΔI_D_*) due to a temperature variation (*ΔT*) induces an error in the measure of the light power (*ΔP_T_*), defined as:

*ΔP_T_ =* (*ΔI_D_/ΔT*)*/R_λ_*   [W/K] (2)

For example, when the temperature presents small variations around 80 °C, applying Equation (2) and considering *R_λ_* values at 450 and 600 nm, the error in the light power measurement is lower than 0.7 pW/K, while at 510 nm wavelength, corresponding to the maximum of R_λ_, the error does not exceeds 0.55 pW/K.

Under large temperature variation (between T_1_ and T_2_), the total error (*ΔP_T,tot_*) in the light power measurement is given by:

*ΔP_T,tot_* = ((*I_D_*(*T_2_*) *− I_D_*(*T_1_*))/*R_λ_*   [W] (3)

**Figure 8:**
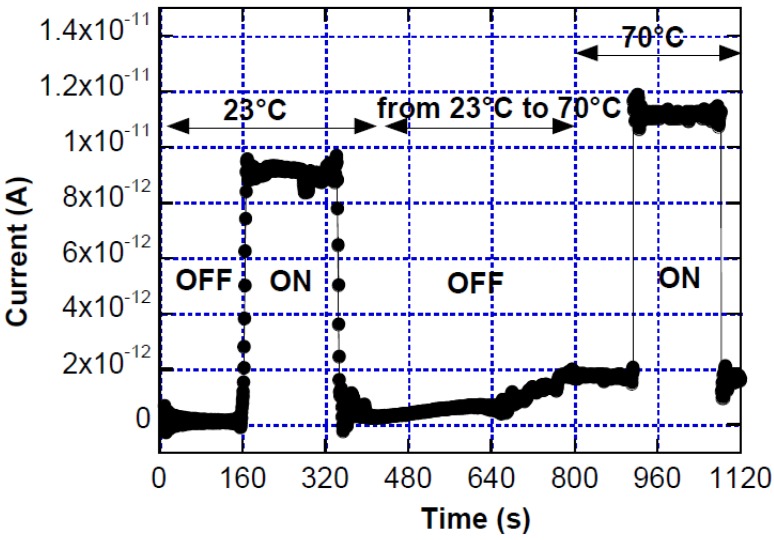
Diode current under two equal light pulses at temperature equal to 23 and 70 °C. ON and OFF refer to turning on and off a light source impinging on the device.

This behavior is illustrated in Figure 8. The two current values, measured under the same monochromatic (465 nm).

Light pulse at 23 and 70 °C, have a difference of 2.1 pA. This value represents the influence of the temperature on the measurement of light intensity. Referring to Equation (3), with T_1_ = 23 °C and T_2_ = 70 °C, and considering a photosensor responsivity (*R*) around 290 mA/W at 465 nm, we calculate a total error in the light intensity measurement equal to 7.25 pW.

### 4.2. Effect of Light Intensity on Temperature Measurement

When the sensor is used to measure the temperature, with the diode biased by a constant forward current, the output signal device is a voltage inversely proportional to the measured temperature. A voltage variation (*ΔV*) induced by a light power variation (*ΔP*) corresponds to an error in the measurement of the temperature (*ΔT_L_*), defined as:

*ΔT_L_ = (ΔV/ΔP)/S_Τ_*   [K/W] (4)

**Figure 9:**
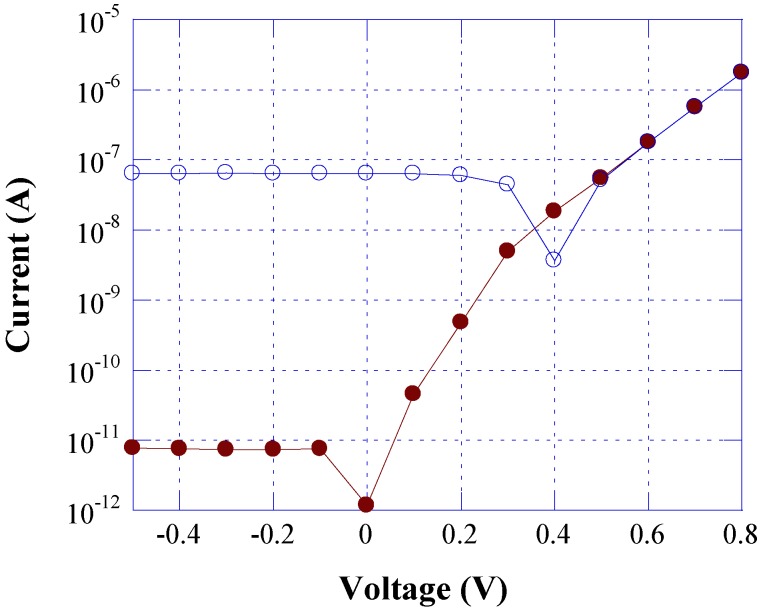
Current-voltage characteristics measured at room temperature in dark conditions (solid symbols) and under monochromatic illumination at 465 nm with light intensity equal to 0.5 µW (open symbols).

This is illustrated in Figure 9, which reports the current-voltage characteristics measured, at stabilized temperature (30 °C), in dark conditions and under 0.5 µW monochromatic illumination at 465 nm. The photocurrent measured in reverse bias is about 10^−7^ A, four orders of magnitude higher than the dark current (10^−11^ A), while in forward bias (above 0.5 V), the two curves are almost superimposed. In particular, we found that, at a bias current of 1 µA, the voltage difference between the two curves, due the light intensity, is equal to 0.85 mV. Taking into account the thermal sensitivity (*S_T_* = 3.2 mV/K) of the a-Si:H diode, we derive that the error in the temperature measurement is equal to 0.5 K at 0.5 µW light intensity. This error can be considered negligible in biological analysis [38], where 0.5 µW is well above the optical power to be detected.

## 5. Conclusions

We have proposed the use of an amorphous silicon p-i-n junction as both temperature and radiation sensor. In particular, we have analyzed the mutual effect of the temperature on light intensity measurements and of the light intensity variation on the accuracy in the temperature measurement. These effects induce very low errors in both light and temperature measurements if the diode is biased in short circuit or in high injection conditions, respectively. These positive characteristics of the structure make the device a very promising candidate as thin film sensor in integrated LOC systems, where optical detection of biomolecules is often required during a heat treatment of the analyte.
